# Reversal of Alopecia Areata Following Treatment With the JAK1/2 Inhibitor Baricitinib

**DOI:** 10.1016/j.ebiom.2015.02.015

**Published:** 2015-02-26

**Authors:** Ali Jabbari, Zhenpeng Dai, Luzhou Xing, Jane E. Cerise, Yuval Ramot, Yackov Berkun, Gina A. Montealegre Sanchez, Raphaela Goldbach-Mansky, Angela M. Christiano, Raphael Clynes, Abraham Zlotogorski

**Affiliations:** a Department of Dermatology, Columbia University, New York, NY, USA; b Department of Pathology, Columbia University, New York, NY, USA; c Department of Dermatology, Hadassah-Hebrew University Medical Center, Jerusalem, Israel; d Department of Pediatrics, Hadassah-Hebrew University Medical Center, Jerusalem, Israel; e Translational Autoinflammatory Disease Section, NIAMS, NIH, Bethesda, MD, USA.; f Department of Genetics & Development, Columbia University, New York, NY, USA; g Department of Medicine, Columbia University, New York, NY, USA

**Keywords:** Alopecia areata, Interferon gamma, JAK inhibitor, CANDLE syndrome, Autoimmune disease, Baricitinib, Gene expression profiling, Autoinflammatory

## Abstract

**Background:**

Alopecia areata (AA) is an autoimmune disease resulting in hair loss with devastating psychosocial consequences. Despite its high prevalence, there are no FDA-approved treatments for AA. Prior studies have identified a prominent interferon signature in AA, which signals through JAK molecules.

**Methods:**

A patient with AA was enrolled in a clinical trial to examine the efficacy of baricitinib, a JAK1/2 inhibitor, to treat concomitant CANDLE syndrome. *In vivo*, preclinical studies were conducted using the C3H/HeJ AA mouse model to assess the mechanism of clinical improvement by baricitinib.

**Findings:**

The patient exhibited a striking improvement of his AA on baricitinib over several months. *In vivo* studies using the C3H/HeJ mouse model demonstrated a strong correlation between resolution of the interferon signature and clinical improvement during baricitinib treatment.

**Interpretation:**

Baricitinib may be an effective treatment for AA and warrants further investigation in clinical trials.

## 1 Introduction

Alopecia areata (AA) is a polygenic autoimmune disease that results in hair loss that ranges in presentation from circular patches on the scalp that can often undergo spontaneous resolution to complete hair loss that may persist for life. There are currently no FDA-approved treatments for AA, and treatment regimens for patients with severe disease are empiric and frequently unsatisfactory. Recent work in human subjects has identified several genes underlying AA (Petukhova et al., 2010, Betz et al., 2015), as well as a prominent interferon (IFN) signature in the AA scalp (Xing et al., 2014), inviting trials targeting this pathway for treatment purposes.

Chronic atypical neutrophilic dermatosis with lipodystrophy and elevated temperature (CANDLE) syndrome (OMIM 256040) is a monogenic autoinflammatory syndrome that was initially described in 2010 (Torrelo et al., 2010). Patients present within the first six months of life with recurrent episodes of fever, periocular erythema and edema, annular violaceous plaques (Supplementary Fig. 1) and lipodystrophy (Torrelo et al., 2010, Ramot et al., 2011, Liu et al., 2012). Alopecia is not reported as a part of the CANDLE phenotype or other immunoproteasome-related disorders (Torrelo et al., 2010, Agarwal et al., 2010, Arima et al., 2011). Mutations have been discovered in *PSMB8*, whose gene product is a component of the immunoproteasome, in some, but not all patients, with CANDLE syndrome (Liu et al., 2012). There are currently no well-established treatments for this rare orphan disease. Whole genome expression analysis of peripheral blood mononuclear cells identified the IFN pathway as being highly dysregulated in these patients (Liu et al., 2012), establishing a possible target for the development of new treatments.

AA and CANDLE syndrome are both characterized by prominent IFN signatures, albeit as a result of different genetic mechanisms. Type I and type II IFNs signal through cell surface receptors that initially activate JAK kinases, specifically JAK1 and either TYK2 or JAK2, respectively. Small molecule JAK inhibitors have been developed and are currently available for myelofibrosis (Verstovsek et al., 2010) and rheumatoid arthritis (RA) (Fleischmann et al., 2012). This class of drugs offers the convenience of being orally bioavailable when compared with biologic inhibitors of IFNs, which require parenteral administration. Here, we describe a patient enrolled in a clinical trial of baricitinib, an oral JAK inhibitor with relative selectivity for JAK1 and JAK2, for CANDLE syndrome who experienced dramatic resolution of his AA.

A 17-year-old man with chronic long-standing AA was enrolled in a clinical trial examining the efficacy of baricitinib for a proposed IFN mediated autoinflammatory syndrome, CANDLE syndrome (NCT01724580). The diagnosis of CANDLE syndrome was based on the typical clinical features of widespread annular violaceous skin lesions and the multisystemic inflammatory characteristics (Ramot et al., 2011) and had been confirmed by finding a homozygous mutation in *PSMB8* (Liu et al., 2012). Prior to the trial, he had been treated for CANDLE syndrome with two courses of intravenous pulses of methylprednisolone and oral methotrexate (10 mg/week). At the time of study initiation, he had been taking oral prednisone at a dose of 12 mg/day.

The patient suffered from a chronic patch-type AA for seven years, which involved mainly his occipital scalp. He had been treated with dithranol cream and minoxidil in the past without improvement of his alopecia. In the time preceding his enrollment in the trial, the patient experienced progression of his disease to an ophiasis pattern, a form of AA that is usually recalcitrant to treatment (Finner, 2011), despite being on an immunosuppressive regimen for CANDLE syndrome.

## 2 Methods

### 2.1 Clinical Studies

Due to the observation that increased STAT-1 phosphorylation and a strong IFN response signature are observed in CANDLE patients (Liu et al., 2012), a treatment trial with the JAK 1/2 inhibitor baricitinib was initiated at the National Institutes of Health (NCT01724580). The patient was enrolled in this study and started to receive once-daily oral baricitinib in September 2012, initially at a dose of 7 mg daily and 6 months later at 7 mg in the morning and 4 mg in the evening, with gradual tapering of oral corticosteroids to 3 mg daily. Informed consent was provided by the patient and his guardians. All forms and protocol were approved by the NIDDK/NIAMS IRB, and the study number on clinicaltrials.gov is NCT01724580.

### 2.2 Animal Studies

We performed three sets of *in vivo* experiments to determine the mechanistic basis for treatment response of AA with baricitinib. Baricitinib was obtained from MedKoo Biosciences (Chapel Hill, NC). The C3H/HeJ graft-recipient mouse model of AA was used for these experiments. C3H/HeJ mice spontaneously develop alopecia at a rate of 10–20% by 6–18 months of age. C3H/HeJ mice that receive skin grafts of alopecic skin from donor alopecic C3H/HeJ mice develop the disease 95–100% of the time by 10 weeks post-transplant. Using the C3H/HeJ grafted mouse model of AA, we first conducted experiments to prevent onset of disease by administering baricitinib at the time of grafting. Briefly, alopecic skin from a C3H/HeJ mouse that spontaneously developed hair loss was grafted onto 8–10 week old C3H/HeJ mice free of disease. At the time of grafting, an osmotic pump (Alzet) that administered approximately 0.7 mg/day of baricitinib or placebo was implanted. Osmotic pumps were changed monthly.

A time-to-event survival analysis for interval censored data was performed. The survival and interval packages in R were used to perform log-rank tests. The hypothesis that the survival distributions are equal in the (n = 10) baricitinib-treated mice and (n = 10) placebo-treated mice is rejected at the 5% level using Sun's score to perform an exact log-rank two-sample test with the p-value of 0.0035.

We then conducted treatment experiments in the setting of established AA in mice, using both systemic delivery and topical delivery of baricitinib. C3H/HeJ recipients of alopecic C3H/HeJ mouse skin were aged at least an additional 12 weeks to allow for near complete alopecia prior to either implantation of osmotic pumps or topical treatment. Osmotic pump administration was conducted in a similar manner as for the prevention experiments. For topical treatment experiments, vehicle control or 0.5% baricitinib was applied topically daily. For these experiments, the R package nparLD was used to test the hypothesis that there exists a treatment by time interaction. A F1–LD–F1 design was employed. The hypothesis of no interaction, *i.e.*, parallel time profiles, is rejected at the 5% level using both the Wald-type statistic and the ANOVA-type statistic with the p-values of 5.80 × 10^− 22^ and 3.74 × 10^− 15^, respectively, for the baricitinib-treated (n = 8) and placebo-treated (n = 8) groups.

At the indicated time points, skin samples were taken for the purposes of immunohistochemical staining and microscopy, immune infiltrate extraction and flow cytometric analysis, and/or RNA expression studies (see Supplementary Materials and methods). Microarray data were deposited in Gene Expression Omnibus with accession number GSE61555.

## 3 Results

### 3.1 Clinical Response to Baricitinib

**Fig. 1:**
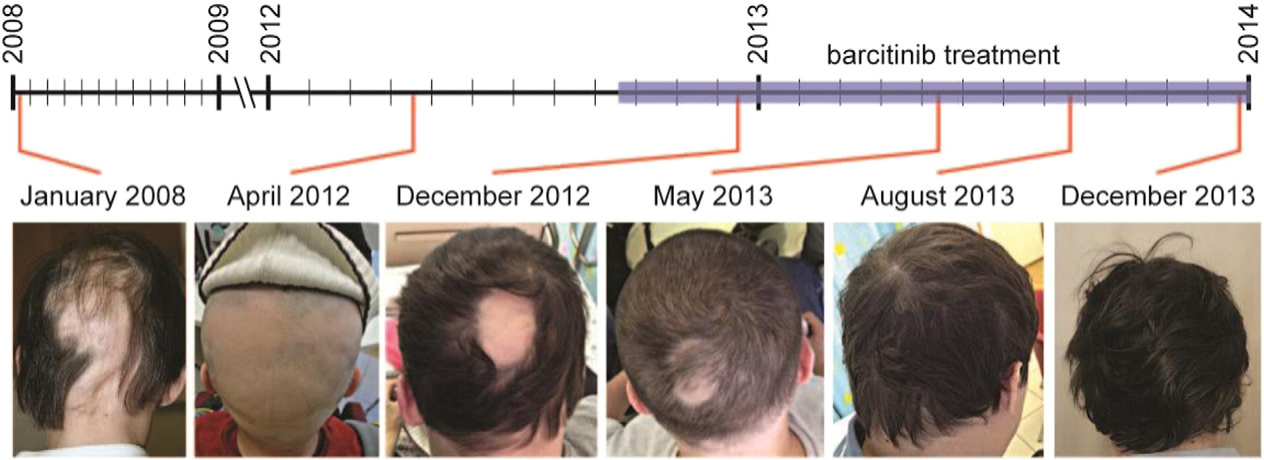
Clinical response to baricitinib in CANDLE patient with AA. Scalp photos of the CANDLE patient with AA prior to and during treatment with baricitinib. Timeline showing the approximate dates that photographs were taken and period in which the patient was being treated with baricitinib.

Soon after starting baricitinib treatment, there was a remarkable improvement in the patient's AA, regressing to only a single patch of hair loss on his occipital scalp three months after starting baricitinib treatment (Fig. 1). There was a steady regrowth of hair on this single patch, until he had complete resolution of hair loss nine months after starting treatment (Fig. 1). With continued baricitinib treatment, his hair regrowth persisted. Today, under baricitinib treatment, the patient has complete hair growth on his scalp, with no signs of recurrence of AA.

### 3.2 Resolution of the IFN Signature

**Fig. 2:**
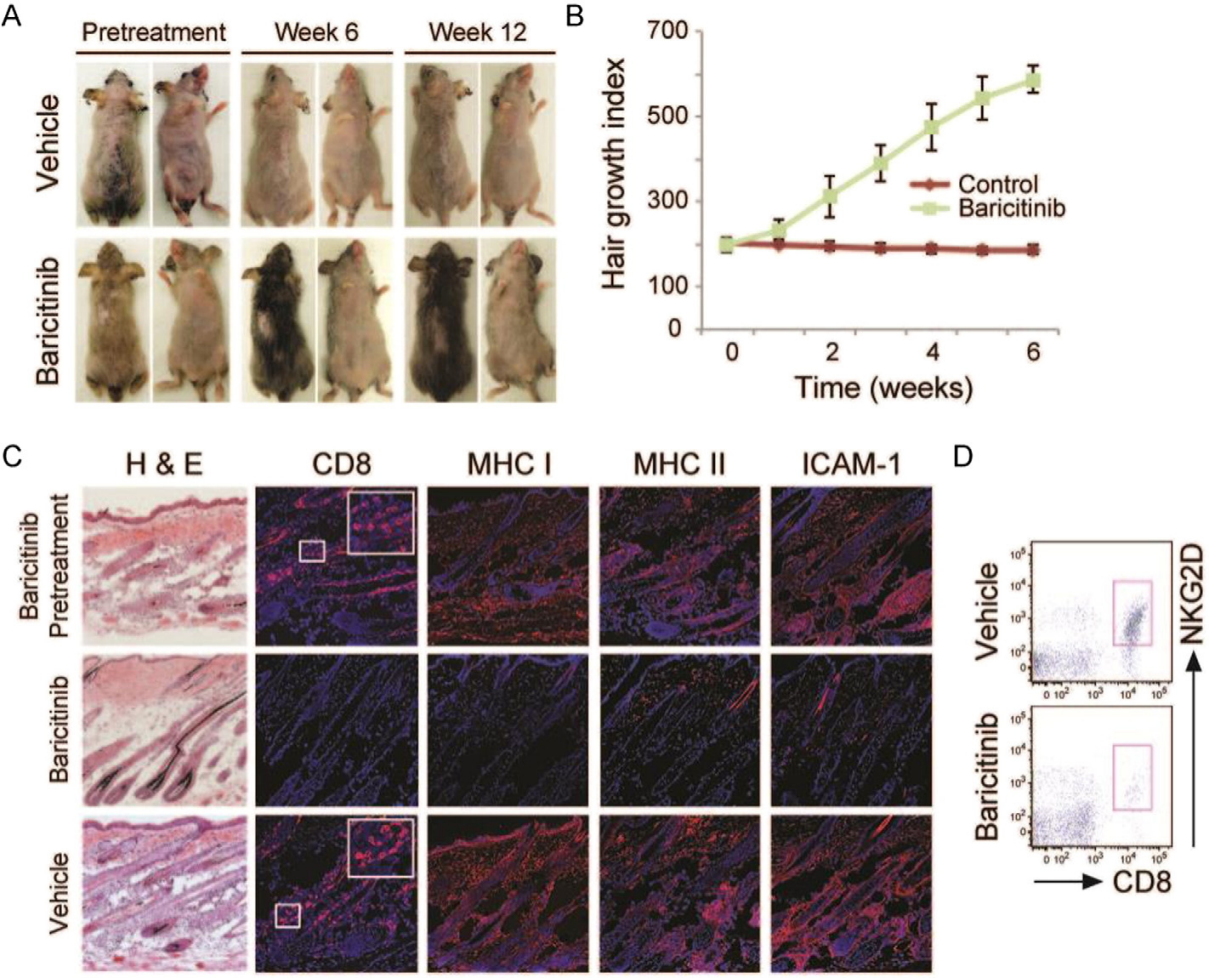
Treatment of AA in C3H/HeJ mouse model. C3H/HeJ graft recipient mice were treated with topical baricitinib or vehicle control after disease establishment. A, Photographs were taken at 12 weeks post-treatment. B, Graph of hair regrowth index in each group over time. C, Skin sections were taken at baseline and at 12 weeks post-treatment and stained with H&E or with antibodies to CD8, MHC class I, MHC class II, or ICAM-1. D, Frequency of CD8^+^NKG2D^+^ cells in treated skin.

Intrigued by the rapid improvement in his AA, we evaluated the effect of baricitinib in a mouse model of AA and conducted mechanistic studies to understand the action of baricitinib. We conducted three sets of *in vivo* experiments to investigate the mechanisms of action of baricitinib for AA. C3H/HeJ grafted alopecic mice were treated with systemically administered baricitinib or vehicle/placebo control either prior to (Supplementary Fig. 2) or following the establishment of alopecia (Supplementary Fig. 3). Furthermore, C3H/HeJ grafted alopecic mice were treated with a topical formulation of baricitinib or vehicle control after the mice developed alopecia (Fig. 2). In all three cases, hair growth was consistently observed in baricitinib-treated mice, compared with no clinical evidence of hair regrowth in vehicle control treated mice (Fig. 2 and Supplementary Figs. 2 and 3). Skin biopsies were taken 12 weeks after the start of treatment and assessed for immune cell infiltration and loss of immune privilege. Baricitinib treated mice exhibited substantially reduced inflammation as assessed by H&E staining, reduced CD8 infiltration, and reduced MHC class I and class II expression when compared with vehicle-control treated mice (Fig. 2). CD8^+^NKG2D^+^ cells, critical effectors of disease in murine and human AA, were greatly diminished in baricitinib treated mice compared with vehicle control treated mice (Fig. 2).

**Fig. 3:**
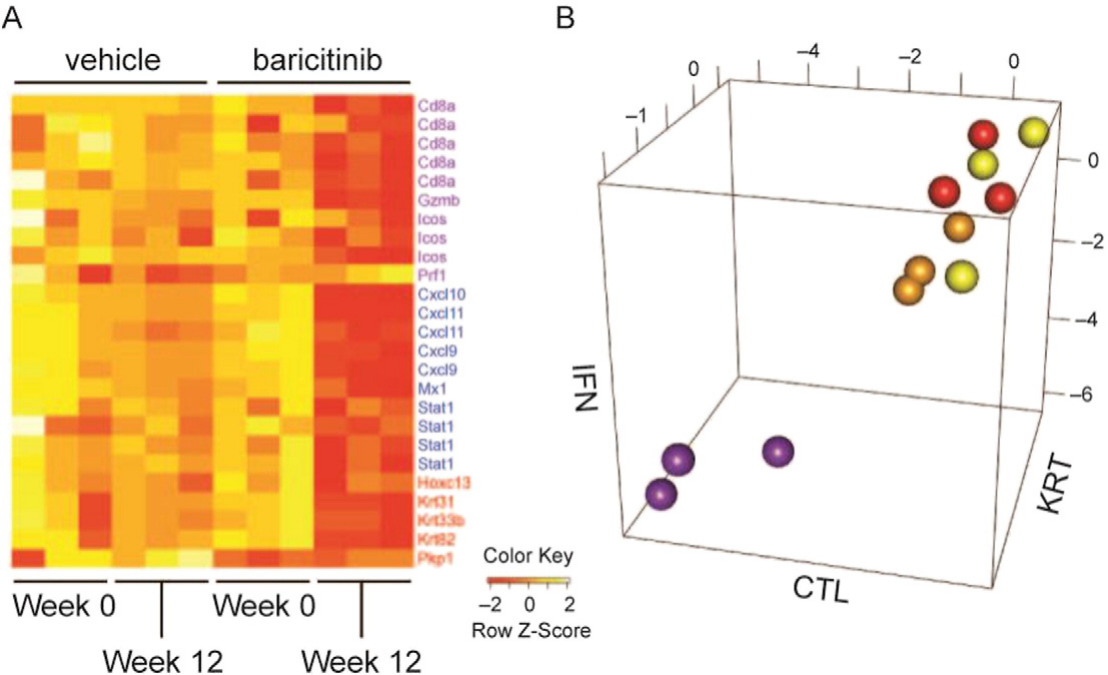
Normalization of IFN signature in baricitinib treated skin. A, Expression of indicated genes in skin and, B, ALADIN score plots, from C3H/HeJ grafted mice treated with topical baricitinib or vehicle control administered after the establishment of disease demonstrating resolution of IFN and CTL scores with baricitinib treatment only. Yellow, control treatment at week 0; orange, control treatment at week 12; red, baricitinib treatment at week 0; purple, baricitinib treatment at week 12.

To define the molecular response to baricitinib, we performed gene expression profiling of treated skin in both the prevention and treatment models. Our previous studies defined an Alopecia Areata Disease Activity Index (ALADIN) biomarker for response to treatment (Xing et al., 2014), which monitors three distinct gene expression signatures, one of which is the IFN response. In all three contexts, we observed rapid normalization of the IFN gene expression signature in response to baricitinib (Fig. 3 and Supplementary Figs. 2 and 3). Both the IFN and cytotoxic T lymphocyte (CTL) components of the ALADIN strongly discriminated between effectively treated mice and mice that did not exhibit disease resolution.

## 4 Discussion

In this study, we report a dramatic clinical response to the JAK inhibitor baricitinib in a patient with longstanding AA. We further define the mechanism of response to baricitinib and resolution of the IFN gene expression signature in the AA mouse model. Recently, three independent studies reported similar clinical responses to JAK inhibitors in patients with AA. We recently showed that the JAK inhibitor ruxolitinib, which also has relative selectivity for JAK1 and JAK2 and is currently approved for myelofibrosis, reversed disease in three AA patients in an open-label clinical trial of oral drug in moderate-to-severe disease (Xing et al., 2014). Secondly, a single patient with alopecia universalis and psoriasis was treated orally with tofacitinib, an FDA approved JAK inhibitor with higher affinity to JAK3, and also showed a clinical response (Craiglow and King, 2014). Lastly, a patient with essential thrombocythemia and alopecia universalis was treated with ruxolitinib and exhibited striking and near-complete hair regrowth after 10 months of treatment (Pieri et al., 2015). Our previous studies using both ruxolitinib and tofacitinib in the C3H/HeJ mouse model of AA demonstrated the molecular underpinnings of the response and resolution of the disease at the cellular and molecular levels (Xing et al., 2014).

Small molecule JAK inhibitors offer several advantages when compared with a therapeutic strategy centered on targeting cytokines with biologics. First, small molecule JAK inhibitors have oral bioavailability, making them more attractive to patients and likely increasing adherence. Second, JAK inhibitors inhibit multiple pathogenic pathways simultaneously, including both type I and type II IFN receptor pathways, both of which appear to be active in AA (Xing et al., 2014, Freyschmidt Paul et al., 2006, Ghoreishi et al., 2010). Notably, small molecule JAK inhibitors may be developed into a topical form in the future, with two published studies examining the efficacy of a topical JAK inhibitor for the treatment of psoriasis (Ports et al., 2013, Punwani et al., 2012). A topical JAK inhibitor may decrease the risk/benefit profile of this class of drugs. Clinical trials examining the efficacy of baricitinib in the context of the compassionate use protocol for CANDLE syndrome as well as for RA are currently underway. Results from a phase IIb clinical trial for baricitinib in RA have been released showing a statistically significant improvement in ACR20 between baricitinib treatment and placebo (Genovese et al., 2012), and phase III trials are currently underway for this indication. AA may represent another potential indication for which baricitinib may be tested in the future. Taken together, the recent reports of dramatic responses to treatment using JAK inhibitors invite broader clinical exploration of the utility of these agents in AA.

## Contributors

AJ, ZD, LX, AMC, RC and AZ contributed to the conception and design of the study. AJ, ZD, LX, YR, YB, GMS, RG-M, and AZ contributed to data collection. AJ, ZD, LX, JEC, YR, YB, GMS, RG-M, AMC, RC, and AZ analyzed and interpreted the data. AJ, AMC, and AZ drafted the report, which was critically revised for important intellectual content by RG-M and RC. All authors approved the final version of the report.

## Declaration of Interests

All authors declare no competing interests.

## References

[bb0035] Agarwal A.K., Xing C., DeMartino G.N. (2010). PSMB8 encoding the β5i proteasome subunit is mutated in joint contractures, muscle atrophy, microcytic anemia, and panniculitis-induced lipodystrophy syndrome. Am. J. Hum. Genet..

[bb0040] Arima K., Kinoshita A., Mishima H. (2011). Proteasome assembly defect due to a proteasome subunit beta type 8 (PSMB8) mutation causes the autoinflammatory disorder, Nakajo–Nishimura syndrome. Proc. Natl. Acad. Sci. U. S. A..

[bb0010] Betz R.C., Petukhova L., Ripke S. (2015). Genome-wide meta-analysis in alopecia areata resolves HLA associations and reveals two new susceptibility loci. Nat. Commun..

[bb0060] Craiglow B.G., King B.A. (2014). Killing two birds with one stone: oral tofacitinib reverses alopecia universalis in a patient with plaque psoriasis. J. Investig. Dermatol..

[bb0055] Finner A.M. (2011). Alopecia areata: clinical presentation, diagnosis, and unusual cases. Dermatol. Ther..

[bb0050] Fleischmann R., Kremer J., Cush J. (2012). Placebo-controlled trial of tofacitinib monotherapy in rheumatoid arthritis. N. Engl. J. Med..

[bb0070] Freyschmidt Paul P., McElwee K.J., Hoffmann R. (2006). Interferon‐γ‐deficient mice are resistant to the development of alopecia areata. Br. J. Dermatol..

[bb0090] Genovese M.C., Keystone E., Taylor P. (2012). 24-Week results of a blinded phase 2b dose-ranging study of baricitinib, an oral Janus kinase 1/Januse kinase 2 inhibitor, in combination with traditional disease modifying antirheumatic drugs in patients with rheumatoid arthritis.

[bb0075] Ghoreishi M., Martinka M., Dutz J.P. (2010). Type 1 interferon signature in the scalp lesions of alopecia areata. Br. J. Dermatol..

[bb0030] Liu Y., Ramot Y., Torrelo A. (2012). Mutations in proteasome subunit β type 8 cause chronic atypical neutrophilic dermatosis with lipodystrophy and elevated temperature with evidence of genetic and phenotypic heterogeneity. Arthritis Rheum..

[bb0005] Petukhova L., Duvic M., Hordinsky M. (2010). Genome-wide association study in alopecia areata implicates both innate and adaptive immunity. Nature.

[bb0065] Pieri L., Guglielmelli P., Vannucchi A.M. (2015). Ruxolitinib-induced reversal of alopecia universalis in a patient with essential thrombocythemia. Am. J. Hematol..

[bb0080] Ports W.C., Khan S., Lan S. (2013). A randomized phase 2a efficacy and safety trial of the topical Janus kinase inhibitor tofacitinib in the treatment of chronic plaque psoriasis. Br. J. Dermatol..

[bb0085] Punwani N., Scherle P., Flores R. (2012). Preliminary clinical activity of a topical JAK1/2 inhibitor in the treatment of psoriasis. J. Am. Acad. Dermatol..

[bb0025] Ramot Y., Czarnowicki T., Maly A., Navon Elkan P., Zlotogorski A. (2011). Chronic atypical neutrophilic dermatosis with lipodystrophy and elevated temperature syndrome: a case report. Pediatr. Dermatol..

[bb0020] Torrelo A., Patel S., Colmenero I. (2010). Chronic atypical neutrophilic dermatosis with lipodystrophy and elevated temperature (CANDLE) syndrome. J. Am. Acad. Dermatol..

[bb0045] Verstovsek S., Kantarjian H., Mesa R.A. (2010). Safety and efficacy of INCB018424, a JAK1 and JAK2 inhibitor, in myelofibrosis. N. Engl. J. Med..

[bb0015] Xing L., Dai Z., Jabbari A. (2014). Alopecia areata is driven by cytotoxic T lymphocytes and is reversed by JAK inhibition. Nat. Med..

