# Supplementary material for: Reversal of Alopecia Areata Following Treatment With the JAK1/2 Inhibitor Baricitinib
Source: eBioMedicine. 2015 Feb 26;2(4):351–5. doi: 10.1016/j.ebiom.2015.02.015 (PMC4486197; doi:10.1016/j.ebiom.2015.02.015)
Supplement: Supplementary file 1 — Supplementary material. [file mmc1.docx]

**SUPPLEMENTARY MATERIAL**

**Table of contents**

Supplementary Materials and Methods 2

Supplementary Figure 1 5

Supplementary Figure 2 6

Supplementary Figure 3 8

Supplementary References 10

**Supplementary Materials and Methods**

**Immunohistochemistry**

Immunohistochemistry and immunofluorescence. 8 μM acetone-fixed frozen mouse or human skin sections were air-dried and stained overnight with the indicated anti-mouse antibodies at 4 degrees C in a moist chamber followed by one hour with the appropriate secondary antibody.

**Flow Cytometric Analysis**

To make a single-cell suspension of mouse skin, fat was removed from the overlying skin in cold PBS and then incubated in collagenase type I (2 mg/ml in PBS) at 32 degrees C for 75 min. After digestion, the skin was minced in RPMI/10% FBS, filtered through a 70-μM cell strainer and centrifuged at 1100g for 5 min. The pellet was resuspended in RPMI/10% FBS, filtered through a 40-μM cell strainer and spun at 400g for 5 min. The pellet was resuspended in FACS buffer (PBS/5% BSA) with DAPI to gate on live cells and staining antibodies as indicated and analyzed on a BD LSR II flow cytometer.

**Gene Expression Profiling**

Gene expression profiling was performed to monitor resolution of the IFN signature in the skin and hair follicles in response to baricitinib. RNA was extracted from flash frozen skin samples using the miRNeasy kit (Qiagen, Inc., Valencia, CA) with on-column DNase digestion using RNase-free DNase kits (Qiagen, Inc.). Reverse transcription, amplification, biotinylation and fragmentation were done using the Ovation RNA Amplification V2 kit and Encore Biotin Kit (NuGen). Labeled, fragmented cDNA libraries were hybridized to GeneChip Mouse Genome 430 2.0 Arrays (Affymetrix), stained with streptavidin-phycoerythrin, and scanned on an HP GeneArray Scanner (Hewlett-Packard Company, Palo Alto, CA).

            Microarray preprocessing and data analysis was performed using BioConductor in R.  Preprocessing, quality control and analysis was performed separately on each of the three experiments: 1) Prevention experiment: five mice treated with baricitinib vs. five mice treated with vehicle with samples taken 12 weeks after initiation of treatment; 2) Topical treatment of mice with established AA: three mice treated with baricitinib vs. three mice treated with vehicle with samples taken at baseline and at 12 weeks after initiation of treatment; and 3) Systemic treatment mice with established AA: three mice treated with baricitinib vs. three mice treated with vehicle with samples taken at baseline and at 12 weeks after initiation of treatment.  The same pipeline was used for each experiment.

Quality control (QC) was performed using the affyanalysisQC package from [http://arrayanalysis.org/](http://arrayanalysis.org/). AffyanalysisQC uses R/BioConductor packages: affy, affycomp, affypdnn, affyPLM, affyQCReport, ArrayTools, bioDistm biomaRt, simpleaffy, yaqcaffy to perform QC within a single script.  RMA normalization^1^ was performed on each of the three experimental groups separately.  One vehicle sample in the prevention experiment, one baricitinib sample at baseline and one baricitinib sample at 12 weeks for the topical experiment, and one control sample at baseline were removed prior to performing downstream analysis because they had failed QC.

**ALADIN scores**

Individual signature IFN, CTL and KRT scores were calculated following procedures used in our previous study.^2^ The sets of genes selected to comprise our IFN, CTL, and KRT signatures were Cd8a, Gzmb, Icos and Prf1 for the CTL signature, Cxcl9, Cxcl10, Cxcl11, Mx1, and Stat1 for the IFN signature, and Hoxc13, Krt31, Krt33b, Krt82, and Pkp1 for the KRT signature.  The scores for all the prevention mice were calculated in relation to the four vehicle mice that had passed QC (Figure 3B); whereas, the scores for the topical treatment and systemic treatment experiments were calculated relative to the vehicle mice of the respective experiments at week zero (Figure 3D and Supplementary Figure 1D). For each experiment, Z-scores were calculated for the probe sets for each of the genes comprising the ALADIN signatures using all samples, and heatmaps were generated using heatmap.3 in R (Figure 3A, 3C, Supplementary Figure 1C).

**Supplementary Figure 1.**


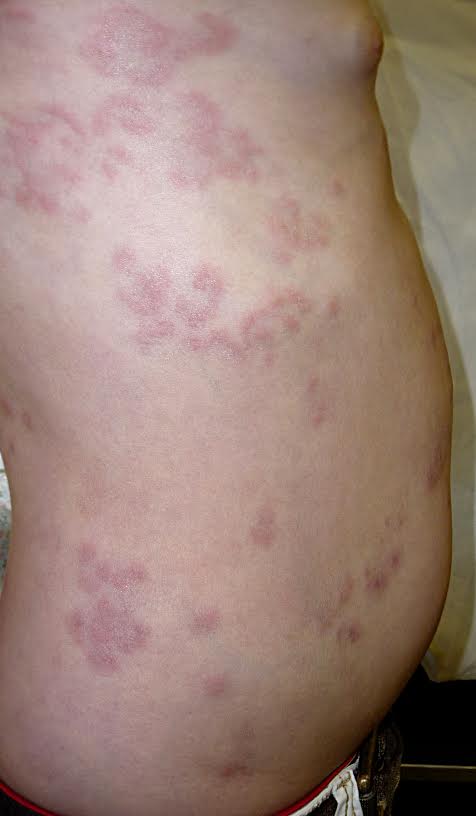


**Supplementary Figure 1. Skin lesions in a patient with CANDLE syndrome.** Shown are the annular erythematous and violaceous plaques seen in a patient with CANDLE syndrome.

**Supplementary Figure 2**

**
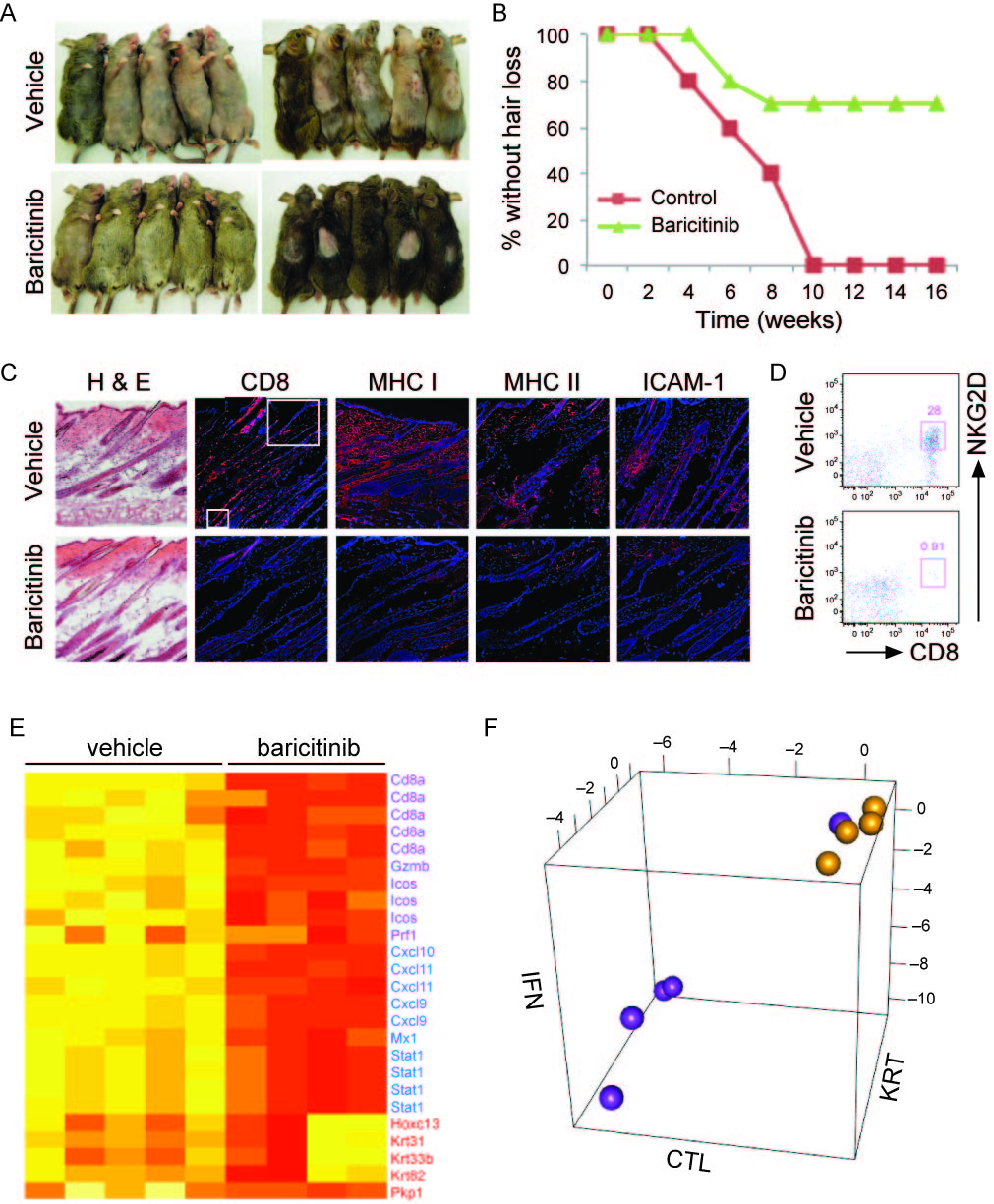
**

**Supplementary Figure 2. Prevention of AA in C3H/HeJ Mouse Model with systemic baricitinib.**

C3H/HeJ grafted mice were treated with baricitinib or placebo control, administered by osmotic pumps implanted at the time of grafting. A, Photographs were taken at 12 weeks post treatment. B, Frequency of mice in each group without hair loss. C, Skin sections were taken at 12 weeks post treatment and stained with H & E or with antibodies to CD8, MHC class I, MHC class II, or ICAM-1. D, Frequency of CD8^+^NKG2D^+^ cells in the skin. E, Expression of indicated genes in skin and, F, ALADIN score plots from C3H/HeJ graft recipient mice treated with baricitinib or vehicle control administered at the time of grafting demonstrating diminished IFN and CTL scores with baricitinib administration compared with vehicle treated controls. Yellow, control treatment; purple, baricitinib treatment.

**Supplementary Figure 3**

**Supplementary Figure 3. Treatment of AA in C3H/HeJ Mouse Model with systemic baricitinib.**

C3H/HeJ grafted mice were treated with baricitinib or placebo control, administered by osmotic pumps after disease establishment. A, Photographs were taken at 12 weeks post treatment. B, Skin sections were taken at 12 weeks post treatment and stained with H & E or with antibodies to CD8, MHC class I, MHC class II, or ICAM-1. C, Expression of indicated genes in skin and, D, ALADIN score plots from C3H/HeJ graft recipient mice treated with baricitinib or vehicle control administered after disease establishment demonstrating resolution of IFN and CTL scores with baricitinib administration only. Yellow, control treatment at week 0; orange, control treatment at week 12; red, bacitinib treatment at week 0; purple, baricitinib treatment at week 12.

**Supplementary References**

1 Irizarry RA, Bolstad BM, Collin F, Cope LM, Hobbs B, Speed TP. Summaries of Affymetrix GeneChip probe level data. *Nucleic Acids Res* 2003; **31**: e15–5.

2 Xing L, Dai Z, Jabbari A, *et al.* Alopecia areata is driven by cytotoxic T lymphocytes and is reversed by JAK inhibition. *Nat Med* 2014; **20**: 1043–9.
